# Blue-Light Fundus Autofluorescence (BAF), an Essential Modality for the Evaluation of Inflammatory Diseases of the Photoreceptors: An Imaging Narrative

**DOI:** 10.3390/diagnostics13142466

**Published:** 2023-07-24

**Authors:** Alessandro Mantovani, Carl P. Herbort, Alireza Hedayatfar, Ioannis Papasavvas

**Affiliations:** 1 Department of Ophthalmology, Valduce Hospital, 22100 Como, Italy; 2 Inflammatory and Retinal Eye Diseases, Centre for Ophthalmic Specialised Care (COS), 1003 Lausanne, Switzerland; cph@herbortuveitis.ch (C.P.H.J.); i.s.papasavvas@gmail.com (I.P.); 3 Eye Research Centre, The Five Senses Institute, Rassoul Akram Hospital, University of Medical Sciences, Teheran 14456 13131, Iran; alireza.hedayatfar@gmail.com; 4 Moorfields Eye Hospital, London EC1V 2PD, UK

**Keywords:** blue-light fundus autofluorescence (BAF), ICGA, MEWDS, APMPPE, MFC, AZOOR, CAR, serpiginous choroiditis

## Abstract

Our purpose is to describe blue-light fundus autofluorescence (BAF) features of inflammatory diseases of the outer retina characterised by photoreceptor damage. BAF from patients diagnosed with secondary and primary inflammatory photoreceptor damage were retrospectively analyzed and compared to other imaging modalities including fluorescein angiography (FA), indocyanine green angiography (ICGA), and spectral domain optical coherence tomography (SD-OCT). Multiple evanescent white dot syndrome (MEWDS), idiopathic multifocal choroiditis (MFC), acute posterior multifocal placoid pigment epitheliopathy (APMPPE), serpiginous choroiditis (SC), and acute syphilitic posterior placoid chorioretinitis (ASPPC), all cases corresponding to secondary photoreceptor diseases caused by inflammatory choriocapillaris nonperfusion, were included and compared to primary photoreceptor disease entities, including acute zonal occult outer retinopathy (AZOOR) and cancer-associated retinopathy (CAR). Both groups showed increased BAFs of variable intensity. In severe cases of APMPPE and ASPPC, BAF also showed hypoautofluorescent areas. In group 1 (secondary diseases) BAF hyperautofluorescent areas were associated with colocalized ICGA hypofluorescent areas, indicating choriocapillaris nonperfusion; whereas in group 2 (primary diseases), no ICGA signs were detected. The associated colocalized areas of hypofluorescence on ICGA in the first group, which were absent in the second group, were crucial to allow the differentiation between primary (photoreceptoritis) and secondary (choriocapillaritis) photoreceptor diseases. BAF patterns in inflammatory diseases of the outer retina can give relevant information on the photoreceptor and RPE involvement, with ICGA being crucial to detect concurring choriocapillaris damage and differentiating the two pathologies.

## 1. Introduction

Fundus autofluorescence (FAF), in particular, blue-light fundus autofluorescence (BAF), is a useful imaging modality for the evaluation of the metabolic status of the outer retina and the retinal pigment epithelium (RPE). BAF has become clinically applicable following the development of technological advancements, including the confocal scanning laser ophthalmoscope (cSLO) and the modified fundus camera (mFC), which allows the detection of low-intensity autofluorescence produced by fluorophores (mainly lipofuscin) at the level of the RPE cells. BAF imaging is a noninvasive, fast, easy, and sensitive diagnostic modality, particularly useful in evaluating inflammatory diseases affecting, either primarily or secondarily, the outer retina. The lesion process can be secondary to chorioretinal interface damage due to inflammatory choriocapillaris nonperfusion, such as in multiple evanescent white dot syndrome (MEWDS), idiopathic multifocal choroiditis (MFC), acute posterior multifocal placoid pigment epiteliopathy (APMPPE), serpiginous choroiditis (SC), and acute syphilitic posterior placoid chorioretinitis (ASPPC), or can be a consequence of a direct insult to the photoreceptors, a so-called primary photoreceptoritis such as acute zonal occult outer retinopathy (AZOOR) and cancer-associated retinopathy (CAR).

## 2. Principle of Blue-Light Fundus Autofluorescence (BAF) Analysis

Using cSLO (Spectralis HRA, Heidelberg Engineering, Heidelberg, Germany), BAF images can be obtained with a blue-light wavelength (excitation 488 nm, emission > 500 nm) [1].

BAF imaging is a technique that allows for the indirect assessment of the RPE and photoreceptor integrity. The BAF signal comes from bisretinoids of lipofuscin accumulated in RPE cells. This material, the major fluorophores in the eye, is a mixture of several bisretinoids (A2E, A2PE…) that are byproducts of the visual cycle; these bisretinoids form primarily in the photoreceptor outer segments and are deposited secondarily in the RPE cell lysosomes during the process of photoreceptor outer segment phagocytosis [2]. BAF images of a normal fundus show a diffuse autofluorescent signal with a dark optic disc (absence of fluorophores) and dark vessels (blocking autofluorescence). In the macular area, the fovea appears hypoautofluorescent because macular pigments (lutein and zeaxanthin) and melanin absorb blue light. Therefore, BAF is not useful to detect retinal alterations in the fovea because of insufficient excitation due to macular pigment blockage [3].

The intensity of the autofluorescent signal is modified by the variation of the types and amounts of fluorophores (lipofuscin and others) on one side and by the absorption of light by macular pigments and the photopigments of the photoreceptor outer segments that act as screens to RPE autofluorescence.

**Figure 1:**
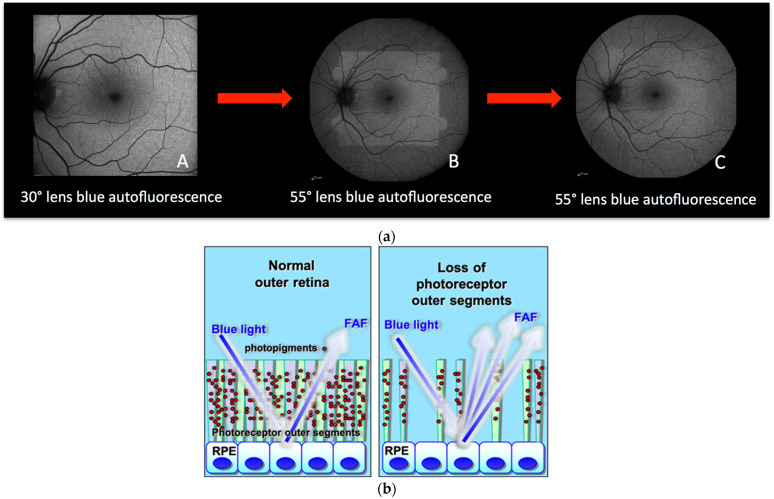
(**a**) **Principles of fundus bleaching**. (**A**) Blue-light fundus autofluorescence acquired with a 30° lens. (**B**) blue-light fundus autofluorescence acquired with a 55° lens readily after the (**A**) acquisition. (**C**) blue-light fundus autofluorescence acquired with the same 55° lens after 30 s of exposure. (**b**) Cartoon explaining hyperautofluorescence in case of loss of photoreceptor outer segments. The lost screen of photopigments allows better detection of the physiological autofluorescence of the RPE.

Blue-light irradiation causes a reduction of the photopigment density in the photoreceptor outer segments (due to the absorption of photons induced by the isomerization of 11-cis retinal to all-trans retinal), causing increased visualization of the autofluorescence signal coming from the RPE. This process is called photobleaching [4,5,6]. An illustrative example shows a normal fundus exposed to blue light coming from a 30° lens (Figure 1a (A)); immediately thereafter, when the same eye is exposed to blue light coming from a wider lens (Figure 1a (B)) this reveals the exhausted photopigment previously exposed to the 30° lens. After some time, the photopigment from the entire retina exposed to blue light from the wide lens is uniformly reduced and the fundus appears bleached (hyperautofluorescent) (Figure 1a (C)). Therefore, in case of damage to the photoreceptors, the consecutive reduction of the photopigment density will lead to a similarly increased visibility of the normal underlying RPE lipofuscin and, hence, increased autofluorescence (hyperautofluorescence).

## 3. BAF in Inflammatory Diseases of the Photoreceptors (Table 1)

In inflammatory diseases of the fundus, BAF analysis is not only useful in the clinical appraisal and follow-up of certain diseases but also contributes further information in addition to other imaging methods to study the lesion process. The inflammatory diseases that cause increased or decreased autofluorescence are mainly those entities for which the inflammatory process involves the choriocapillaris-RPE complex and the outer retina. The BAF pattern may be different in the same disease entity depending on the severity and/or stage of the disease. Basically, these diseases can cause either hyperautofluorescence or hypoautofluorescence. On one side, the more striking alteration is the presence of areas of hyperautofluorescence that can be caused either (1) by photoreceptor damage or (2) by RPE damage causing accumulation of “non-digested” fluorophores. Usually, in this situation, the hyperfluorescent spots are brighter.

**Table 1:** Characteristics and explanation of hyperautofluorescence changes in diseases affecting the photoreceptors.

	Mechanism of Hyperautofluorescence	Co-Localized ICGA Hypofluorescent Areas	After Photobleaching	Type of Hyperautofluorescence
**Severe choriocapillaris nonperfusion**	Accumulation of fluorophores in RPE	**+++**	No change	Bright
**Secondary photoreceptor damage (caused by choriocapillaris nonperfusion)**	“Window defect” (loss of photopigment in the outer segments) + (possible rare areas of accumulation of fluorophores)	**+++**	Disappearance of hyperautofluorescence contrast (with possible few areas with nonerased more bright FAF)	Soft with possible few areas of brighter hyper-FAF
**Primary photoreceptor diseases**	Window defect (loss of photopigment in the outer segments)	**No**	The disappearance of hyperautofluorescent lesions	Soft

**Figure 2:**
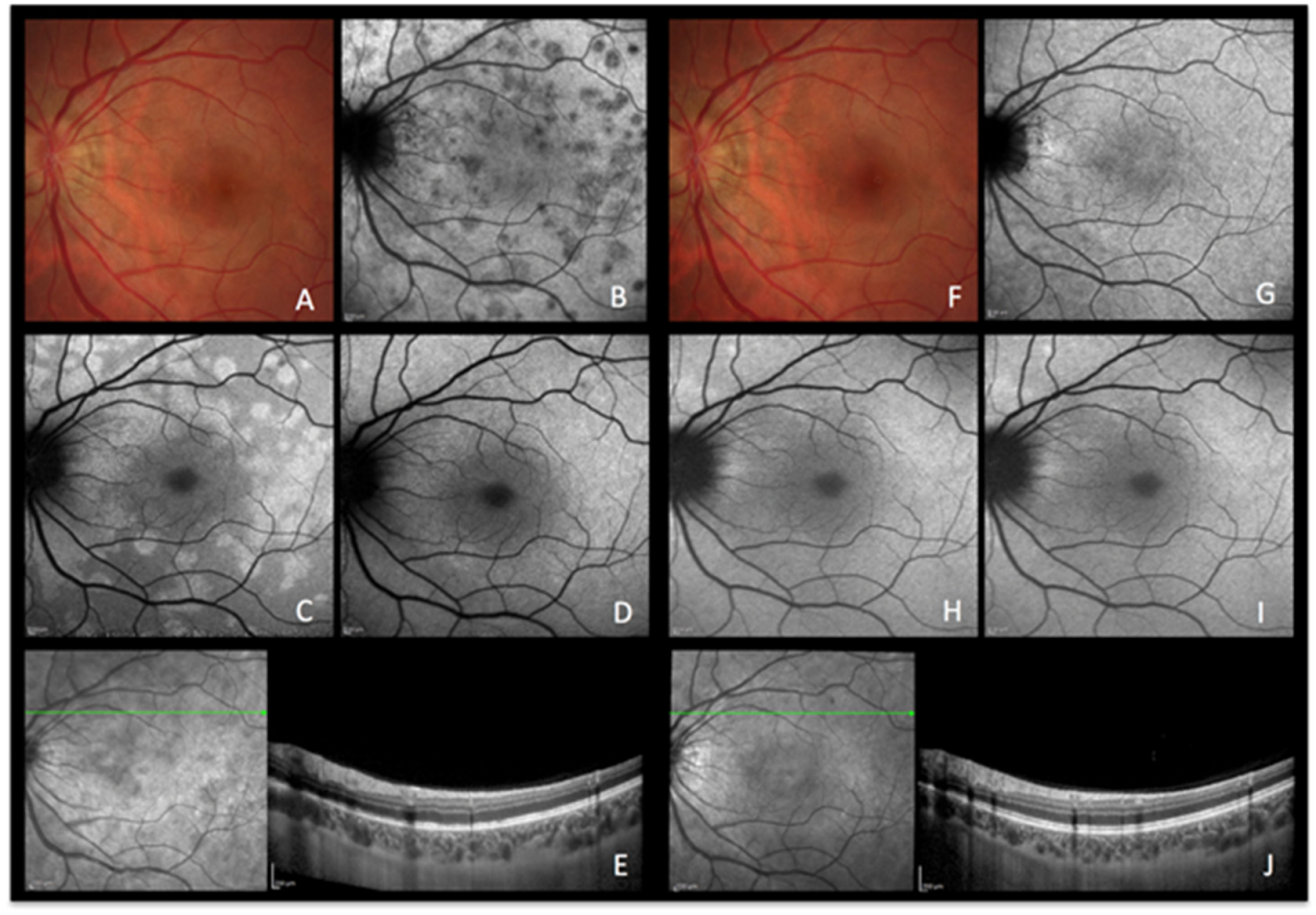
**MEWDS imaging during active and inactive disease.** (**A**–**E**), baseline acquisitions. (**F**–**J**) acquisitions after recovery. (**A**,**F**) colour fundus photography. (**B**,**G**) late indocyanine green angiography; numerous hypofluorescent areas of nonperfusion (**B**) disappearing after reperfusion (**G**). (**C**,**H**) blue-light autofluorescence; numerous hyperautofluorescent areas colocalising with ICGA hypofluorescent areas and resolving in the healed stage of the disease. (**D**,**I**) blue-light autofluorescence after bleaching. (**E**,**J**) infrared and optical coherence tomography showing loss of photoreceptor outer segments (**E**) with reconstitution in the healed stage (**J**).

**Figure 3:**
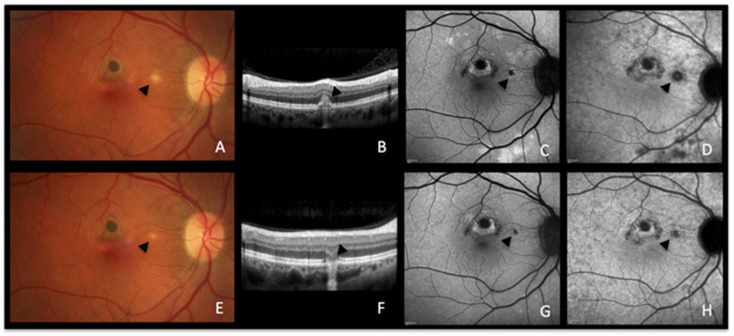
**MFC imaging during active and inactive disease.** (**A**–**D**) baseline acquisitions. (**E**–**H**) acquisitions after recovery. (**A**,**E**) colour fundus photographs showing a new fluffy lesion (black arrowhead) (**A**) evolving to an inactive scar (**E**). (**B**,**F**) optical coherence tomography showing the morphology of the active lesion (**B**, black arrowhead), recovering in the convalescent phase (**F**, black arrowhead) with a remaining scar. (**C**,**G**) blue-light autofluorescence showing hyperautofluorescent areas in the active stage (**C**) resolved in the convalescent treated stage (**G**). (**D**,**H**) late indocyanine green angiography showing hypofluorescent areas in the active stage (**D**), resolved in the convalescent treated stage (**H**).

In the case of photoreceptor damage, in acute disease, areas of hyperautofluorescence are detected that, after the photobleaching process (brief illumination with blue light) become isoautofluorescent [6,7] (Figure 2 and Figure 3). In this situation, spectral domain-OCT (SD-OCT) findings show disruptions in the hyperreflective bands of the ellipsoid zone (EZ) and the interdigitation zone (IZ) that correspond to the photoreceptors. These disruptions are the result of photoreceptor outer segment loss or disorganization. For these reasons (photobleaching and SD-OCT findings), the areas of hyperautofluorescence, present in the initial BAF images, are due to better visualization of the physiological RPE autofluorescence because of the loss of the photopigment screen represented by photoreceptors and not because of an increase of fluorophores. In fact, the normalization of the BAF images occurs at the moment of restoration of the photoreceptor layer, documented by SD-OCT. The mechanism related to the lack of photopigment can be explained by two types of lesion processes. On one side, photoreceptors can be damaged secondarily with the main mechanism being an RPE impairment since it is the main site for 11-cis retinal regeneration in the visual cycle, mostly as a consequence of inflammatory choriocapillaris non-perfusion-producing ischaemia on the RPE–photoreceptor complex. ICGA, which is the gold standard to detect choriocapillaris nonperfusion, shows hypofluorescent dark areas that are more evident in the late phase of the angiography. The conditions where this mechanism is most preeminent are MEWDS, MFC, APMPPE, SC, and ASPPC. On the other hand, the outer retina can be primarily affected by direct damage to the photoreceptors. In the latter case, choriocapillaris circulation is intact and ICGA fluorescence is normal and conserved. These conditions are called primary photoreceptoritis and comprise entities such as AZOOR and CAR. BAF hyperfluorescent signs can be produced both by primary damage to the photoreceptors and by secondary ischaemic damage caused by choriocapillaris nonperfusion. To differentiate between these two mechanisms, ICGA is crucial as it can detect choriocapillaris nonperfusion even when it is minimal, as in MEWDS. Moreover, the photobleaching process brings the healthy photoreceptors to exhaustion, thus increasing the autofluorescence of the unaffected areas which now become isoautofluorescent to the hyperautofluorescent affected areas.

In some cases where choriocapillaris nonperfusion is severe, the ischaemic process can be very pronounced. In very acute stages, the RPE becomes metabolically less active and accumulates fluorophores, which is seen as bright hyperautofluorescence. As the ischaemia persists, it leads to the loss of RPE cells and scarring that causes concomitant areas of hypoautofluorescence, such as in severe cases of APMPPE and SC. In mild cases of choriocapillaris nonperfusion, such as MEWDS, mild APMPPE and mild MFC, RPE is more resistant to the ischemia than the photoreceptors and the mechanism of hyperautofluorescence is explained by the loss of photopigment in the outer segment or accumulation of fluorophores in RPE. Table 1 summarizes the hyperautofluorescence findings in inflammatory diseases affecting the photoreceptors primarily or secondarily.

## 4. Results/Cases

### 4.1. BAF-Signs in Secondary Photoreceptor Diseases (Illustrative Examples)

#### 4.1.1. Multiple Evanescent White Dot Syndrome (MEWDS) Figure 2

##### Case 1

MEWDS is a rare posterior uveitis characterized by numerous, faint, “evanescent” white dots seen in the posterior pole and the midperiphery. Foveal granularity is a common finding and often represents the only detectable sign of the disease, as the white dots have often already disappeared when the patient decides to consult [8].

A healthy 26-year-old woman presented with a three-day history of photopsias in her left eye. The colour fundus photography lacked the typical foveal granularity while faint whitish lesions were only barely visible (Figure 2A). On ICGA images, multiple hypofluorescent areas, more clearly visible on the late frames, were seen during the acute phase of the disease, a sign of choriocapillaris hypoperfusion (Figure 2B) [9]. In correspondence with ICGA hypofluorescent areas, BAF showed hyperautofluorescence that disappeared after photobleaching and OCT showed disruption of the ellipsoid zone (loss of photoreceptor outer segments) (Figure 2E). In the convalescent phase, fundus photography, BAF, and ICGA images normalized (Figure 2F–I). SD-OCT follow-up scans through the affected locations showed recovery with a normal aspect of the hyperreflective bands of the outer retina (Figure 2J). The disappearance of BAF hyperautofluorescent spots after photobleaching indicates that these spots resulted from photoreceptor damage (secondary to choriocapillaris nonperfusion). Due to the acute onset and relatively short temporary course of the disease, the ischaemic process did not possess enough intensity and duration to cause RPE damage and subsequent abnormal fluorophore accumulation.

#### 4.1.2. Idiopathic Multifocal Choroiditis (MFC) Figure 3

##### Case 2

MFC is a choriocapillaritis with a moderate involvement of the choriocapillaris, characterized by chorioretinal lesions which often have a recurrent course with subclinical novel recurrent lesions clearly identified by ICGA. Sometimes, the initial episode resembles MEWDS. The diagnosis is often made when there is a recurrence and new active ICGA-detected lesions appear along with old chorioretinal scars. The overlapping of the two diseases (MFC and MEWDS) has been mentioned in the literature [10,11].

A 51-year-old woman consulted, complaining of recurrent photopsias in her right eye. The colour fundus photograph, together with SD-OCT (Figure 3A,B), BAF, and ICGA (Figure 3C,D) at baseline showed an active lesion (Figure 3A, black arrowhead), coexistent with older punched-out atrophic chorioretinal lesions. In the recurrent phase of the disease, active novel chorioretinal lesions have the same aspect as MEWDS lesions on multimodal imaging, indicating that the lesion process is most probably comparable. The similar BAF and ICGA features of MEWDS (Figure 2B,C) and MFC (Figure 3C,D) confirm that both entities are due to choriocapillaritis. In the convalescent phase, the colour fundus photograph, SD-OCT, BAF, and ICGA showed the atrophic changes of the healed inflammatory focus (Figure 3E–H).

#### 4.1.3. Acute Posterior Multifocal Placoid Pigment Epitheliopathy (APMPPE) Figure 4a–c

##### Cases 3a and 3b

APMPPE is a rare bilateral posterior uveitis featured by the abrupt development of multiple placoid lesions affecting the posterior pole as well as the midperiphery. It has been established that choriocapillaris inflammatory nonperfusion and consecutive ischaemia of the outer retina with frequent macular involvement is the driving cause of the phenotypical presentation of the disease; while mild ischaemia is entirely reversible, severe cases may cause RPE cell death and consecutive chorioretinal scarring.

**Figure 4:**
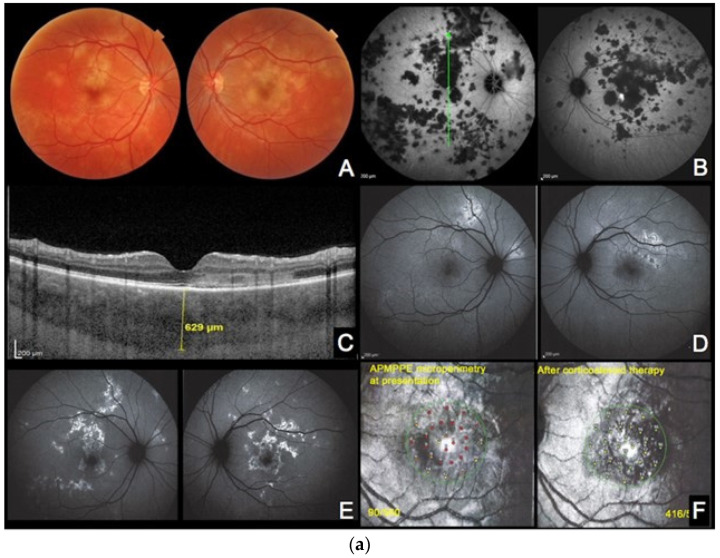

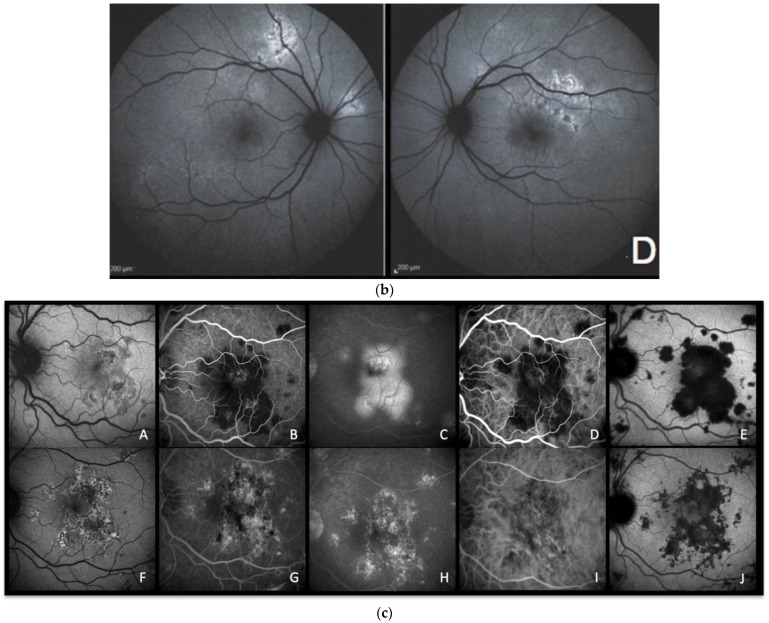
(**a**) **APMPPE during active and inactive disease.** (**A**–**D**), baseline acquisitions. (**E**,**F**) acquisitions after recovery. (**A**) colour fundus photography showing bilateral yellowish placoid lesions. (**B**) late-phase indocyanine green angiography showing extensive hypofluorescent areas of choriocapillaris nonperfusion. (**C**) optical coherence tomography (scan position as shown in (**B**)) showing oedematous thickening and disorganisation of the outer retina and thickening of whole choroid (629 µm) (**D**,**E**) fundus autofluorescence, at the hyperacute stage with minimal hyperautofluorescent areas yet (**D**), followed by bright hyperautofluorescent areas due to RPE damage at a later stage (**E**). (**F**) macular mesopic microperimetry (OS) showing a severe decrease of retinal sensitivity at presentation (90/560), recovering after treatment to 416/560 (bottom far right). (**b**) **APMPPE (enlargement of Figure 4a (A,D))** These BAF frames were obtained at the hyperacute stage of APMPPE when nonperfusion was already widespread (Figure 4a (**A**,**B**)) before lesions causing RPE damage were established and apparent on BAF in all areas of nonperfusion. (**c**) **APMPPE during active and inactive disease.** (**A**,**E**) baseline acquisitions. (**F**–**J**) acquisitions after recovery. (**A**,**F**) blue-light autofluorescence is quasi-absent at the early stage due to profound ischaemia-induced changes (**A**) and followed thereafter by bright hyperautofluorescent areas indicating RPE damage (**F**); (**B**,**G**) represent early phase fluorescein angiography (FA) in acute disease showing hypofluorescence due to choriocapillaris nonperfusion (**B**), while in inactive disease, hyperfluorescent areas indicate window defect due to atrophy (**G**). (**C**,**H**) represent late-phase FA showing in the acute phase pooling due to permeability of retinal vessels induced by severe outer retinal ischaemia (**C**), while in inactive disease, FA shows the same hyperfluorescent window-defect atrophic areas. (**D**,**E**,**I**,**J**) represent indocyanine green angiographic frames showing hypofluorescence due to choriocapillaris nonperfusion, early and late, in the acute phase (**D**,**E**) and hypofluorescence due to atrophy, early and late, in the inactive phase (**I**,**J**).

**Case 3a.** (Figure 4a,b) A 29-year-old man was sent to emergency for hyperacute bilateral chorioretinitis. He was under treatment for a febrile illness with upper respiratory symptoms. He was complaining of photopsias and subjective scotomas. Fundus examination showed bilateral placoid lesions in the posterior pole and midperiphery (Figure 4a,c (AA)). ICGA showed bilateral extensive geographic areas of hypofluorescence, indicating severe choriocapillaris nonperfusion (Figure 4a (AB)). SD-OCT sections through hypofluorescent areas on ICGA showed disruption, blending, and thickening of the outer retinal layers, as well as thickening of the whole choroid. (Figure 4a (AC)).

Surprisingly, the areas of hyperautofluorescence were comparatively rare compared to the extended choriocapillaris nonperfusion shown by ICGA, which was explained by the fact that there was no loss of photoreceptors but disorganisation and oedema of the IZ and EZ (Figure 4a (AD)). The BAF map consisted of small, well-demarcated patches of bright hyperautofluorescence resulting from fluorophore accumulation in metabolically impaired RPE cells (Figure 4b—enlargement of Figure 4a (AD)).

Three days later, after a negative infectious workup for syphilis, and as the lesions had progressed, oral corticosteroids were started. BAF frames performed 8 days later showed remarkably increased scattered areas of bright hyperautofluorescence. This can be explained by the prolongation of the ischaemic process which, in turn, causes further fluorophore accumulation in ischaemia-induced metabolically impaired RPE cells (Figure 4a (AE)). In one parafoveolar area in the left eye, there was even hypoautofluorescence speaking to the loss of RPE cells. Under corticosteroid therapy, most of the visual-field defects and defects on the microperimetry recovered (Figure 4a (AF)).

**Case 3b** (Figure 4c) A 33-year-old man presented with sudden onset of scotomas in his left eye. The BAF was characterized by a central area of iso- to hypofluorescence. (Figure 4c (CA)) Some curvilinear spots of bright hyperautofluorescence were also detectable in the BAF image that surrounded the profoundly hypoautofluorescent islands, which represented, respectively, the partially and totally damaged RPE cells. Ischaemia-induced hypoautofluorescence corresponded to the underlying affected choriocapillaris lobules, as shown by hypofluorescence on early FA (Figure 4c (CB)) and early to late ICGA images (Figure 4c (CD,CE)). The late FA image depicted the typical late hyperfluorescent pooling (Figure 4c (CC)) due to severe outer retinal ischaemia-induced compensatory inner retinal vessel leakage. During recovery, BAF showed typical areas of bright hyperautofluorescence spots (fluorophore accumulation in metabolically damaged RPE cells) and hypofluorescence (loss of RPE) (Figure 4c (CF)), secondary to variable grade of RPE residual damage, whereas FA (Figure 4c (CG,CH)) and ICGA (Figure 4c (CI,CJ)) showed the appearance of RPE–outer retinal scars in early and late frames, respectively.

#### 4.1.4. Serpiginous Choroiditis Figure 5a–c

##### Cases 4a and 4b

Serpiginous choroiditis is a bilateral recurrent autoimmune choriocapillaritis that can sometimes be induced by cross-reactivity to previous mycobacterium tuberculosis exposure.

**Figure 5:**
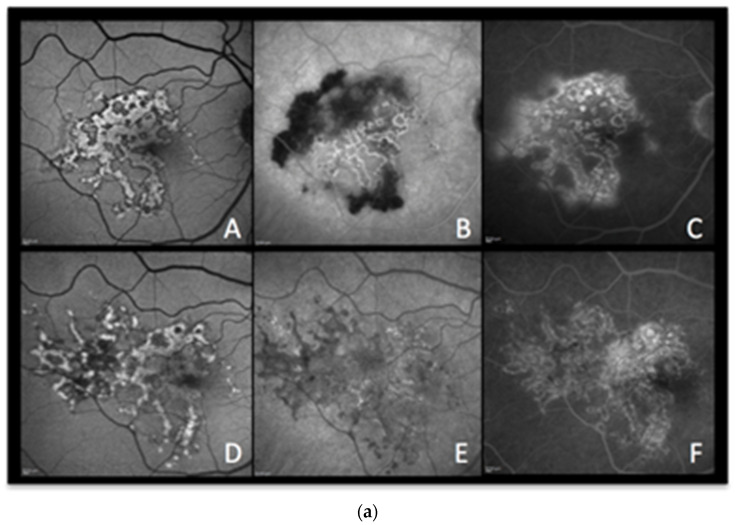

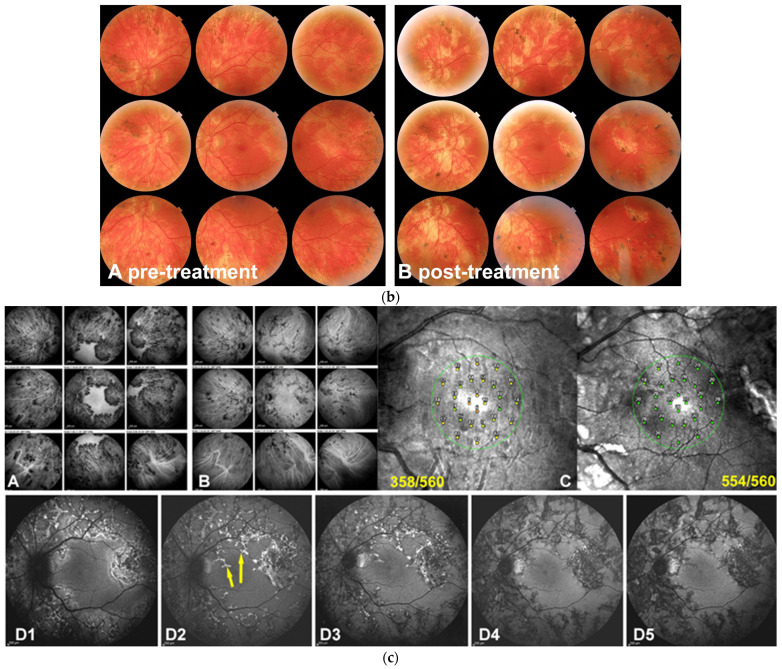
(**a**) **Serpiginous choroiditis (tuberculosis-related) imaging during active and inactive disease.** (**A**–**C**) baseline acquisitions. (**D**–**F**) acquisitions in the convalescent phase. (**A**,**D**) blue-light autofluorescence showing bright autofluorescence due to ischaemia-induced RPE damage during acute and subacute phases. (**B**,**E**) late-phase indocyanine green angiography showing profound hypofluorescent nonperfusion in the progressing areas in active disease (**B**) with recovery of some of the nonperfused areas in the convalescent stage (**E**). (**C**,**F**) late-phase fluorescein angiography showing diffuse exudation in the acute phase (**C**) and window-effect showing the atrophic areas in the convalescent stage (**F**). (**b**) **Serpiginous patient 4b OS.** Fundus panorama pictures at presentation pre treatment (**A**) and the end of follow-up post-treatment (**B**). Apart from increased pigmentation, minimal changes can be observed. (**c**) **Case 4b, Tuberculosis-related serpiginous chorioretinitis OS. Evolution of lesions on ICGA and BAF.** At presentation (**A**), widespread ICGA hypofluorescent areas all over the fundus except in the central macula, indicating either chorioretinal atrophy or inflammatory choriocapillaris nonperfusion; three years after dual immunosuppressive and antituberculous therapy a large proportion of hypofluorescent areas due to nonperfusion have recovered with the remaining hypofluorescent areas representing atrophic areas. (**B**) Although the macula was spared, microperimetry showed decreased retinal sensitivity that recovered completely after treatment (**C**). BAF (**D1**–**D5**) was the most sensitive modality to follow the lesion process with numerous lines of BL-FAF bright hyperautofluorescence due to the accumulation of lipofuscin products from progressing destruction of photoreceptors (**D1**). Two months later (**D2**), a slight decrease in bright hyperautofluorescent rims but an apparition of newly affected areas (yellow arrows). Four months later (**D3**), a substantial decrease of bright hyperautofluorescence at the border of lesions. One year later (**D4**), hyperautofluorescent rims have almost completely disappeared, indicating complete arrest of the progression of lesions and confirmed 6 months later (**D5**).

**Case 4a.** (Figure 5a) A 64-year-old woman was admitted for recurrent photopsias and metamorphopsias in the right eye. The BAF image at the first visit (Figure 5a,b (AA)) showed mixed autofluorescence with overall sharp margins and some marginal areas with a more blurred hyperautofluorescence. The ICGA image revealed those areas to be the front of inflammatory activity (Figure 5a (AB)) that leaked in FA late images (Figure 5a (AC)). The patient underwent a full uveitis workup and, after the positive result for tubercular immunoreactivity tests (QuantiFERON-TB interferon release assay test), she was started on antitubercular treatment and oral steroids. In the follow-up visits, those areas seen on BAF, ICGA, and FA shared the same pattern with the rest of the lesions, suggesting resolution of the active inflammation with residual scarring (Figure 5a (AD), Figure 5a (AE) and Figure 5a (AF), respectively).

**Case 4b.** (Figure 5b,c) (Bilateral comparable involvement but only left eye will be shown) A male patient aged 36 years was referred for a bilateral serpiginous choroiditis with a positive TB IGRA test because he did not respond to systemic prednisone therapy associated with antituberculous antibiotic therapy. At presentation, the central vision was full (1.0 OU). There was no clinical anterior inflammation but a few bilateral vitreous cells. Fundus examination revealed a bilateral serpiginoïd choroiditis in the midperiphery along the vascular arcades and beyond sparing both central maculas (Figure 5b) Microperimetry showed a decrease of retinal sensitivity bilaterally (Figure 5a,c (CC)).

ICGA was characterized by widespread areas of hypofluorescence at presentation, indicating either choriocapillaris nonperfusion or chorioretinal atrophy, with only the central maculas showing normal fluorescence (Figure 5c (CA)).

BAF showed bright hyperautofluorescent rims at the level of progressing lesions around the preserved macular region, produced by the accumulation of lipofuscin products within damaged RPE cells generated by progressing fundus lesions (Figure 5c (CD1)). After the introduction of dual multiple antituberculous and multiple immunosuppressive treatment, the evolution of the bright hyperautofluorescent areas gave a precious follow-up indication about the evolution of lesions and their response to therapy. Indeed, at presentation, BAF localized exactly the active lesions with damaged but surviving RPE cells (Figure 5c (CD1)). After 2 months of aggressive dual immunosuppressive and antituberculous treatment, lesions continued to progress (Figure 5c (CD2), yellow arrow) and started to progressively diminish at 4 months and 12 months (Figure 5c (CD3,CD4)) with quasi-absence of bright BAF spots at 18 months, indicating that the disease was under control (Figure 5c (CD5)). In such cases, BAF appears as the most sensitive modality to follow lesions and orient therapeutical intervention.

In parallel, the ICGA hypofluorescent areas of choriocapillaris nonperfusion progressively regained normal fluorescence with time, under treatment, while areas of complete chorioretinal atrophy at presentation remained hypofluorescent representing scarred areas (Figure 5c (CB)). Microperimetry showed recovery of central retinal sensitivity with a score improving from 358/560 to 554/56 in the left eye (Figure 5a,c (CC)) and from 330/560 to 424/560 in the right eye (not shown).

#### 4.1.5. Acute Syphilis Posterior Placoid Chorioretinitis (ASPPC) Figure 6

##### Case 5

ASPPC can be associated with posterior syphilis and corresponds to a para-infectious immune inflammatory choriocapillaritis.

**Figure 6:**
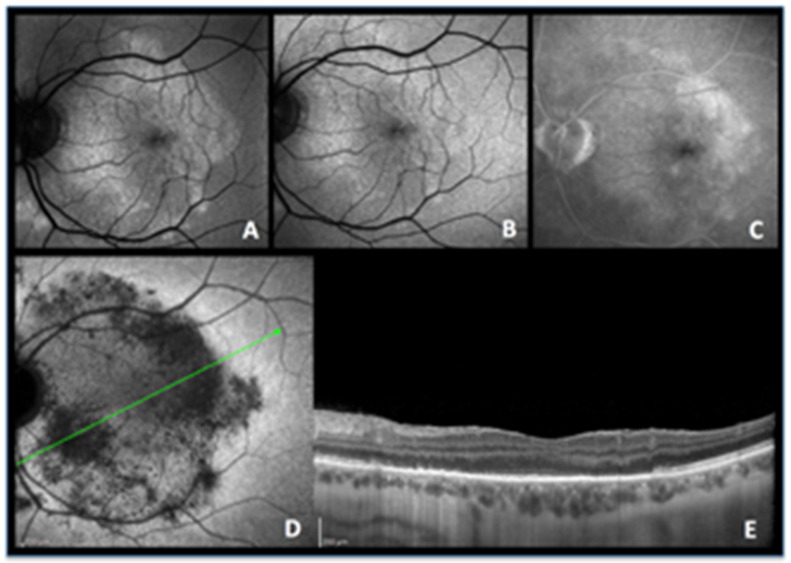
**ASPPC, active disease.** (**A**) blue-light autofluorescence showing a vast area of hyperautofluorescence which is bleached by blue-light irradiation (**B**). (**C**) late fluorescein angiography showing some exudation. (**D**) indocyanine green angiography shows that there is vast hypofluorescence corresponding to extensive nonperfusion. (**E**) The corresponding optical coherence tomography image shows extensive loss of photoreceptor outer segments following choriocapillaris nonperfusion shown on ICGA, explaining the colocalized hyperautofluorescence.

A 49-year-old man presented with a four-day history of scotomas in his left eye. BAF imaging showed a vast, round zone of hyperautofluorescence in the posterior pole (Figure 6A), diming significantly after photobleaching (Figure 6B), that colocalized to the fluorescein angiography hyperfluorescent zone (Figure 6C) and to indocyanine green angiography hypofluorescence (Figure 6D). On OCT, the area of loss of the EZ and IZ (Figure 6E) rigorously corresponded to the area of late hypofluorescence in ICGA. The reduction of hyperautofluorescence to isofluorescence after photobleaching signified that the choriocapillaris non-perfusion-induced ischaemia mainly resulted in photoreceptor damage.

#### 4.1.6. Summary of Secondary Photoreceptor Disease (Choriocapillaritis)

MEWDS (Figure 2), MFC (Figure 3), APMPPE (Figure 4a–c) and SC (Figure 5a–c) are all choriocapillaritis diseases for which the etiology or triggers have not been identified. They involve preferentially young adults and, in a substantial proportion of cases, a flu-like illness precedes the ocular episode, hinting at a viral infection as a possible trigger. For other para-infectious choriocapillaritis entities such as ASPPC and tuberculosis-related serpiginous choroiditis, the trigger that elicits the choriocapillaritis is known. The choriocapillaritis is not caused by the infectious agent itself but is an immune mechanism triggered by infectious agents [12]. Indeed, it was shown for ASPPC that corticosteroid therapy suppressed choriocapillaritis, clearly pointing towards an immune mechanism and not towards the direct implication of the infectious agent [13].

When the level of involvement of choriocapillaris nonperfusion involves larger portions, the severity of the choriocapillaris ischaemia causes a relevant damage to the RPE and outer retina, such as in APMPPE (Figure 4a) [14], and, in serpiginous choroiditis (Figure 5) [15,16], BAF can be decreased within the active lesions being sometimes visible on the border of active or progressing lesions, reflecting RPE/photoreceptor damage. In these conditions, during the convalescent phase of the disease, bright BAF is often present in the centre of scarred lesions, indicating the accumulation of cellular fluorophore debris and reactive hypertrophy/hyperplasia of RPE [17].

## 5. BAF Signs in Primary Photoreceptoritis (Illustrative Cases)

When the primary affected structure is the outer retina with normal fluorescence on ICGA frames, indicating conserved choriocapillaris circulation, the condition can then be called primary photoreceptoritis, comprising acute zonal occult outer retinopathy (AZOOR) [18] and carcinoma-associated retinopathy (CAR) [19]. The mechanisms leading to primary photoreceptoritis are much less known, though inflammatory factors certainly play a role. As a consequence of the primary photoreceptor damage, the transient reduction of the photopigments results in better visualization of the autofluorescent signal.

CAR is an autoimmune retinopathy due to cross-reacting autoantibodies against retinal antigens, mainly recoverin, that are believed to be responsible for photoreceptor damage.

### 5.1. Acute Zonal Occult Outer Retinopathy (AZOOR) Figure 7

#### Case 6

A 56-year-old lady was sent to our centre for a scotoma in her right eye that had first been noted several years prior to her visit. At that time, electrophysiology showed a diffuse alteration of rod function. Two years prior to her visit, her doctors had started an immunosuppressive treatment for her systemic autoimmune disease.

**Figure 7:**
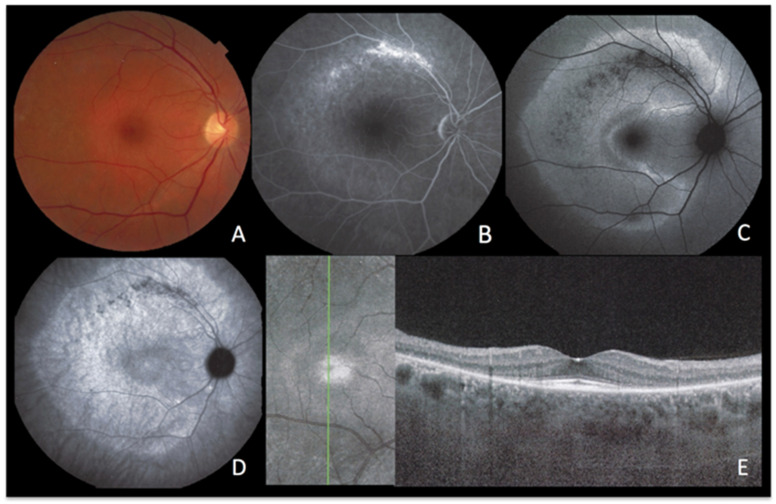
**AZOOR imaging during active disease.** (**A**) Colour Fundus Photography. (**B**) late-phase fluorescein angiography. (**C**) blue-light autofluorescence. (**D**) late-phase indocyanine green angiography. (**E**) infrared imaging with optical coherence tomography. The fundus shows a pale discoloured halo around the fovea that retains a normal colour (**A**) due to loss of photoreceptor photopigment. FA (**B**) shows the same halo of discreet hyperfluorescence due to photopigment loss and an area of bright hyperfluorescence (window effect) along the superior temporal arcade due to chorioretinal atrophy [dark on ICGA (**D**) and on FAF (**C**)]. ICGA (**D**) shows preserved choriocapillaris (except in the arciform area of chorioretinal atrophy along the superior temporal arcade) with increased fluorescence in the area of loss of the screen of photopigments, which also explains fundus hyperautofluorescence (**C**). SD-OCT (**E**) shows the loss of photoreceptor outer segments.

Colour fundus photography (CFP) showed a pale ring around the fovea (Figure 7A). FA showed a faint hyperfluorescent ring due to photoreceptor impairment (loss of photopigment), corresponding to the pale halo on CFP (Figure 7B), along with an arch-like formation of brighter fluorescence along the superior arcade (window defect). BAF demonstrated a striking C-shaped pattern of a well-demarcated area of hyperautofluorescence around the fovea (loss of photoreceptor outer segments) enclosing the same limited area of sharp hypoautofluorescence along the upper temporal arcade resulting from chorioretinal atrophy (Figure 7C).

Late ICGA frames showed the same striking pattern of hyperfluorescence corresponding to the C-shaped hyperautofluorescent areas on BAF, except in the atrophic area along the superior temporal arcade, which was hypofluorescent, corresponding to hypoautofluorescence on FAF and hyperfluorescence on FA (Figure 7D). The hyperfluorescence on ICGA indicated conserved choriocapillaris which was more fluorescent because of loss of photoreceptor outer segments. The SD-OCT image indeed confirmed the extensive loss of photoreceptors in the involved areas. (Figure 7E). Except for the described small area of chorioretinal atrophy, the vast majority of the involved fundus displayed an intact choriocapillaris with overlying outer retinal damage. The preserved outer retinal layers in the foveal area (as seen on SD-OCT) explain the negligible changes seen on FA, BAF, and ICGA in the fovea. This is the natural behaviour of most AZOOR cases which spare the central macula.

This case illustrates clearly that the pathology in AZOOR is primarily at the level of the photoreceptors and not due to choriocapillaritis, which can, however, be involved secondarily during disease progression.

### 5.2. Cancer-Associated Retinopathy (CAR) Figure 8

#### Case 7

A 56-year-old woman with no personal or family history of eye diseases presented with bilateral decreased vision and reported photopsias for 2 weeks. 30 years before, she was treated for uterine cancer.

**Figure 8:**
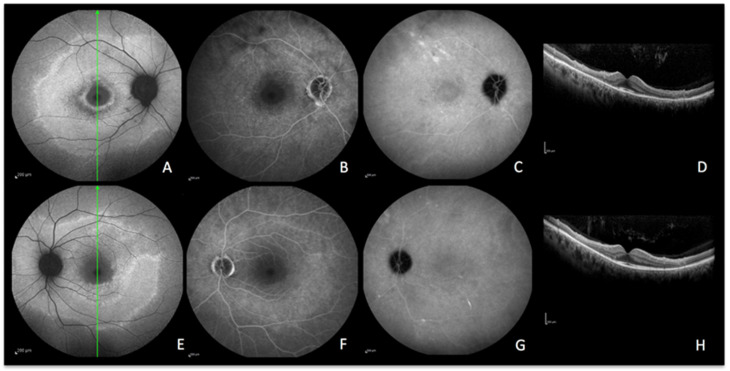
**CAR imaging during active disease.** (**A**–**D**) right eye. (**E**–**H**) left eye. A and E, blue-light autofluorescence showing hyperautofluorescence OD and OS. (**B**,**F**) late-phase fluorescein angiography showing faint hyperfluorescence due to loss of photopigment. (**C**,**G**) late-phase indocyanine green angiography showing absence of hypofluorescence meaning intact choriocapillaris OD and OS. (**D**,**H**) optical coherence tomography showing loss of photoreceptors OD and OS with preservation of the macular area.

Bilateral BAF imaging showed hyperautofluorescence around the arcades and in the peripapillary area (Figure 8A,E) with a corresponding loss of the EZ and IZ with preservation of the macular area in SD-OCT images (Figure 8D,H).

While FA showed faint hyperfluorescence (Figure 8B,F), ICGA did not demonstrate hypofluorescence, indicating preserved choriocapillaris and the absence of choriocapillaris nonperfusion (Figure 8C,G). The increased BAF and the loss of the EZ and IZ on SD-OCT clearly documented the loss/disruption of the photoreceptor layer.

## 6. Discussion

Since the availability of ICGA in the mid-1990s, imaging access to the choroid opened investigations into the numerous diseases of the choroid, allowing their global evaluation and their classification into stromal choroiditis and choriocapillaritis. ICGA is crucial for choriocapillaritis as it is able to precisely determine the pattern of inflammatory choriocapillary nonperfusion. The development of multimodal imaging of chorioretinal diseases, in particular SD-OCT and BAF, further allowed for the determination of the pathophysiology of the different conditions, as well as guides the differentials, especially in infectious conditions [20,21]. BAF hyperautofluorescence in outer retinal diseases was explained thanks to SD-OCT which showed loss of the outer segments of the photoreceptors in the areas of subdued, soft hyperautofluorescence. The areas of a bright hyperfluorescence indicate the impaired function of the RPE cell that becomes congested with fluorophores that cannot be metabolized any longer, unveiling areas of active choroidal inflammation among inactive scars [22]. On the other hand, the absence of autofluorescence indicates either a loss of RPE cells in chorioretinal scars or blockage from overlying blood or other masking lesions. In any case of altered BAF, a multimodal imaging approach with the simultaneous interpretation of BAF and ICGA helps the clinician to distinguish secondary outer retina involvement due to choriocapillaris damage, from primary photoreceptor involvement, as in AZOOR and CAR. In fact, with the availability of multiple imaging modalities, it is possible to demonstrate that photoreceptors can be the primary site of disease without choriocapillaris involvement. To add on, BAF can also represent a useful stand-alone imaging modality in certain posterior uveitis, such as in typical MEWDS cases without any concurring disease, where it can even replace ICGA, as it is noninvasive and, therefore, easy to repeat for closer follow-ups.

## 7. Conclusions

In posterior uveitis, changes in BAF help to suspect outer retinal damage and, depending on the extension, duration, and severity of choriocapillaris and outer retina involvement, the autofluorescence patterns can vary. Hyperautofluorescence is determined by limited changes in RPE and photoreceptors loss, while hypoautofluorescence is the result of wider damage involving the RPE structure.

ICGA is key to establishing the functional status of the choriocapillaris and RPE underlying the damaged retina and, in turn, to distinguish between secondary and primary damage of the photoreceptors.

## Data Availability

Please refer to the corresponding author.
